# IGFBP-2 Signaling in the Brain: From Brain Development to Higher Order Brain Functions

**DOI:** 10.3389/fendo.2019.00822

**Published:** 2019-11-22

**Authors:** Shumsuzzaman Khan

**Affiliations:** Department of Neurobiology and Physiology, School of Medicine, Zhejiang University, Hangzhou, China

**Keywords:** IGFBP-2, IGF-IR, CNS, growth and repair, learning and memory, information processing

## Abstract

Insulin-like growth factor-binding protein-2 (IGFBP-2) is a pleiotropic polypeptide that functions as autocrine and/or paracrine growth factors. IGFBP-2 is the most abundant of the IGFBPs in the cerebrospinal fluid (CSF), and developing brain showed the highest expression of IGFBP-2. IGFBP-2 expressed in the hippocampus, cortex, olfactory lobes, cerebellum, and amygdala. IGFBP-2 mRNA expression is seen in meninges, blood vessels, and in small cell-body neurons (interneurons) and astrocytes. The expression pattern of IGFBP-2 is often developmentally regulated and cell-specific. Biological activities of IGFBP-2 which are independent of their abilities to bind to insulin-like growth factors (IGFs) are mediated by the heparin binding domain (HBD). To execute IGF-independent functions, some IGFBPs have shown to bind with their putative receptors or to translocate inside the cells. Thus, IGFBP-2 functions can be mediated both via insulin-like growth factor receptor-1 (IGF-IR) and independent of IGF-Rs. In this review, I suggest that IGFBP-2 is not only involved in the growth, development of the brain but also with the regulation of neuronal plasticity to modulate high-level cognitive operations such as spatial learning and memory and information processing. Hence, IGFBP-2 serves as a neurotrophic factor which acts via metaplastic signaling from embryonic to adult stages.

## Introduction

The insulin-like growth factor (IGF) system is a mitogenic protein family that includes IGF-I and IGF-II and six binding proteins (IGFBP-I–IGFBP-6) and is involved in functions from embryonic growth to cell differentiation to homeostasis, mostly mediated by IGF-lR (1–3). In the central nervous system (CNS), this system performs various functions, including neurotropic, neuromodulatory, and neuroendocrine ones during embryonic, and postnatal development. This system is responsible for the regulation of brain mass homeostasis and neural stem cell differentiation and proliferation (4–9). In addition, IGFs regulate the growth and differentiation of fetal neurons in culture, and, in addition to promoting synapse formation; IGFs stimulate myelin synthesis and regulate neuronal cytoskeletal protein synthesis with modulation of the expression of immediate early genes (10–13). IGF-system also regulates metabolic functions, including glucose uptake in glial cells, and demonstrates neuromodulatory activities, including enhanced serotonin biosynthesis, inhibition of norepinephrine reuptake by neurons, and maintenance of the Na^+^/K^+^ pump in synaptosomes (14). IGF-ll is involved with memory enhancement and consolidation (7). IGF-l signaling yield through the phosphoinositide 3-kinase (PI3K)-AKT and RAS-extracellular signal-related kinase (ERK) cascades via IGF-IR (15) while the IGF-IIR induces signaling through G proteins that activate protein kinase C (PKC) and phospholipase C (PLC), ultimately regulates Ca^2+^ homeostasis (16).

In the systemic circulation, the bioavailability, and functions of IGF-l and IGF-ll are mainly regulated by six high-affinity IGFBPs. Though IGFBPs share common sequence homology and functions to regulate IGFs actions, individual proteins (IGFBP-1-6) each have their own unique properties and functions. It has been suggested that overexpression of IGFBPs could be a good model to elucidate the physiological functions of individual IGFBPs (17, 18). IGFBP-2 is most abundant in the CSF (19) and highly expressed in the developing brain. In terms of structure, IGFBP-2 consists of three regions: the N-terminal cysteine-rich region, the middle or linker region, and the C-terminal cysteine-rich region (20, 21). Interestingly, IGF-I and IGF-II bind with the N-terminal and C-terminal regions of IGFBP-2 indicates that the high-affinity interactions are mediated by those terminal regions (22, 23). NMR analysis showed that C-terminal region is more prominent in binding with IGF-I and IGF-II and therefore prevent their binding with IGF-IR (24). IGFBP-2 binds with IGFs to control their bioavailability and localization. Thus, IGFBP-2 may act as an important modulator of IGFs signaling in the CNS. Interestingly, local activity-dependent release of IGFs in the CNS also reported. Thus, most important biological functions of either IGFBP-2 or IGFs would be mediated independently in the CNS but passively by IGFBP-2/IGFs complex. Binding of IGFBP-2 to glycosaminoglycans depends on IGFBP-2/IGFs complex (25), however, opposite effect of IGFBP-2 and IGF-I was observed in follicle-stimulating hormone (FSH)-dependent aromatase expression. Thus, deepening on certain physiological conditions, cell type, and tissue/organ, in order to interact with cell surface or ECM, IGFBP-2 may or may not require binding with IGFs. IGFBP-2 also possesses a nuclear localization signal (NLS) sequence within its linker region to interact with Importin-α (26).

**Figure 1:**
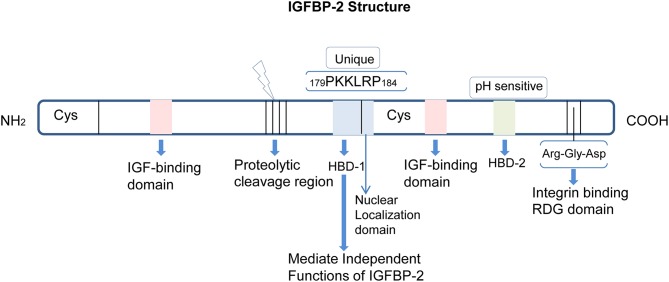
Structure and domains of IGFBP-2. IGFBP-2 consists of N-terminal and C-terminal regions linked by a linker domain. Both the N- and C-terminal have one IGF-binding domain. There is a proteolytic cleavage site between the two terminals. The HBD-1, HBD-2, and RDG domains are located in the cysteine-rich C-terminal region. The nuclear localization domain is adjacent to the HBD1 domain.

In addition, IGFBP-2 possesses several binding domain and among them the two important binding domains (27, 28) are: the Arginine, Glycine, and Aspartate domain (RGD) and the heparin-binding domain (HBD).The RGD domain consists of the sequence Arg-Gly-Asp at peptide position 265RGD267 (the numbering used excludes the signal peptide sequence) in humans and at 246RGD248 in the rat (20, 29) and other species, at C-terminus region (30), and it is responsible for recognition by integrin receptors (31). The RGD domain of IGFBP-2 mediates association with integrins on the cell membrane (32–35). The HBD domain of IGFBP-2 has a consensus sequence for glycosaminoglycan, and, this recognition via HBD is represented by the sequence 160PKKLRP166 in rat IGFBP-2 and 179PKKLRP184 in human IGFBP-2 protein (36). Moreover, the HBD domain in IGFBP-2, the HBD-1, is unique and is not present in other IGFBPs. HBD-1 mediates the binding of IGFBP-2 to cell surface receptor proteins such as tyrosine phosphatase β (37). An additional pH-sensitive heparin-binding site, the HBD-2, shares sequence similarity with other HBD containing IGFBPs such as IGFBP-3 and IGFBP-5 and has been located within the C-terminal region and thyroglobulin type-1 domain of IGFBP-2 [Figure 1; (38)].

Thus, IGFBP-2 is a multifunctional protein that contains IGF-, integrin-, and heparin-binding domains (39), indicating complex regulation and functions of this protein. It has been reported that IGFBP-2 can be cleaved proteolytically, and the smaller fragments that contain HBD still possess the biological activity necessary to bind with extracellular matrix proteins (ECM) and to bind with low affinity to IGF-l (39, 40). Khan et al. (41) showed that the HBD domain of IGFBP-2 independently activates glutamate receptors that translate into enhanced information processing in the hippocampus in a cell type-specific manner, which is crucial for cognitive development at early life in rodents. In addition, IGFBP-2 binding to cell surface proteoglycan in rat olfactory bulb is solely mediated by HBD and not by RGD peptides (39). Mice lacking the RGD domain in IGFBP-2 showed growth impairment compared to wild-type control mice (42) and decreased chondrocyte differentiation, proliferation, and apoptosis in enchondral ossification (43). Moreover, IGFBP-2 enhanced glioma tube formation via RGD domain by interacting with integrin α-5 or β-1 (44). Thus, biological activities of IGFBP-2 can be independent of their abilities to bind to IGFs and these IGF-independent actions can be mediated via the HBD or RGD domains of IGFBP-2.

Transgenic mice overexpressing IGFBP-2 showed reduced body weight without affecting organ mass except in spleen, suggesting that IGFBP-2 is a negative regulator of postnatal growth (45). On the contrary, IGFBP-2 knockout mice revealed no change in body weight, including no effect on brain size or morphology. Targeted inactivation of the *IGFBP2* gene in mice resulted in only subtle phenotypic changes, potentially due to functional compensation by other IGFBPs (46). In addition, IGFBP-1, IGFBP-3, and IGFBP-4 levels were increased in IGFBP-2 knockout mice, suggesting the existence of a compensatory mechanism in the absence of IGFBP-2 (46), a further extension to the complexity of IGFBP-2 functions. In rat brain olfactory bulb, IGFBP-2 has been detected with proteoglycans in the interstitial space and on cell membranes of the mitral cell layer (39, 47). Via the HBD domain, IGFBP-2 can bind to different glycosaminoglycans: chondroitin-4 and−6 sulfate, keratan sulfate, aggrecan, heparin, vitronectin, laminin, collagens, and fibronectin, a probable mechanism for IGFBP-2 to bind with ECM proteins prior to complex formation with IGF-I/II (25, 39, 48, 49), suggesting IGFs-independent functions of IGFBP-2 mediated by the HBD domain. Interestingly, proteolytic degradation of IGFBP-2 can occur on the neuronal surface (39), may be the probable mechanism by which the HBD may directly bind with IGF-IR or other receptors (AMPAR, NMDAR, GABAR) on neurons (41, 50). IGFBP-2 also exerts its functions within cells since it has been detected within the nucleus, nuclear surface, and cytoplasm (51). Thus, IGFBP-2 has both extracellular and intracellular functions. Unlike other IGFBPs, IGFBP-2 does not glycosylate. IGFBP-2 is phosphorylated at serine 106 at an analogous position out of the HBD domain, but phosphorylation levels are significantly lower than that of other IGFBPs (e.g., IGFBP-5) (52). Besides the regulation of IGFs functions (53), several IGFBPs have been shown to reveal IGF-independent functions, especially in bone formation (39). However, in the CNS, IGF-independent functions of IGFBP-2 mostly remain unknown.

## Expression Pattern Of IGFBP-2 In CNS

In the postnatal period, IGFBP-2 is most abundant in the CNS (53) and the second most abundant IGFBP in circulation (39). During early fetal development, IGFBP-2 is expressed in the neuroepithelium of the telencephalon, whereas in later stages of fetal development, it is concentrated in astroglial cells adjacent to IGF-I expressing projection neurons in the retina, cerebellar cortex, and sensory relay centers such as thalamus (53). In the embryonic CNS, IGFBP-2 is expressed in three types of non-neuronal tissues, such as the epithelium of the choroid plexus which is responsible for the production of CSF; the floor plate which is responsible for neuronal outgrowth from spinal cord commissural neurons; and the infundibulum which is responsible for the production of the posterior pituitary (54).

After birth, IGFBP-2 levels significantly decrease in glial cells, although levels have been reported to increase with age (55). Moreover, IGFBP-2 in the CSF is thought to be locally synthesized by the epithelium of the choroid plexus, rather than derived from plasma crossing the blood-brain barrier (BBB) (56), although it may possible that IGFBP-2 may cross the BBB (1). Interestingly, in humans, central and peripheral expressions of IGFBP-2 differ considerably. The highest concentrations of plasma IGFBP-2 were shown at birth. IGFBP-2 levels declined with age during the early years of life in humans but had little change after puberty (57). On the other hand, Ho and Baxter showed a positive correlation between IGFBP-2 levels in serum with age in healthy individual (58). Moreover, IGFBP-2 levels were found no change throughout the puberty aged 7–20 (59), while Blum et al. showed lower levels of IGFBP2 at puberty (60). Similarly, IGFBP-2 is also differentially expressed in the CNS. Neonatal rat CSF contained more IGFBP-2 than adult CSF. IGFBP-2 found in CSF may also be synthesized locally by glia and neurons (19). In addition, IGFBP-2 differed substantially between serum and plasma (57). Males showed slightly higher values of IGFBP-2 in serum than females (57). Moreover, it may also be possible that IGFBP-2 expression also differs in humans and rodents. During mouse adulthood, IGFBP-2 and IGF-II levels in the CNS and CSF remain elevated (61, 62), in contrast to IGF-I levels which generally decline throughout the CNS following development, except in the olfactory bulb, suggesting that IGFBP-2 may regulate the function of IGF-II in the CNS.

**Figure 2:**
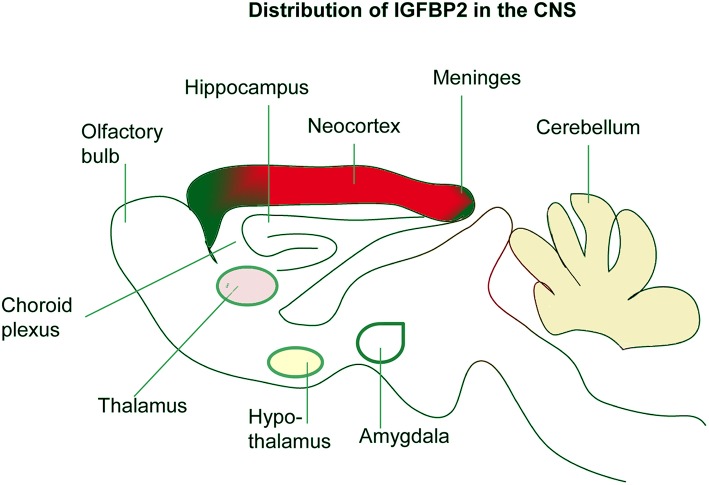
Localization of IGFBP-2 in the brain. IGFBP-2 is abundantly expressed in the hippocampus, neocortex, olfactory bulb, and cerebellum. IGFBP-2 is also found in the meninges, choroid plexus, thalamus, hypothalamus, and amygdala.

Developing rats with malnutrition showed decreased levels of IGF-I in circulation, but IGF-I and IGF-IR in the hypothalamus and cerebellum were increased with the concomitant reduction of IGFBP-2 in the hypothalamus (63). This suggests independent functions of IGFBP-2 in the CNS to maintain brain mass homeostasis. It is also reported that none of the major sites of IGFBP-2 mRNA accumulation in the CNS contain detectable IGF-II mRNA (54), further suggesting IGFBP-2 mediated developmental processes may be independent of IGFs binding. IGFBP-2 is also expressed by Bergmann glia in the cerebellum and Muller cells and astrocytes of the retina (55). Interestingly, at later stages of brain development, IGFBP-2 expression is directly proportional to the expression of IGF-I (55) in the cerebellum, retina, and developing sensory networks (Figure 2). However, in the developing hippocampus and neocortex, IGFBP-2 expression, particularly in astrocytes, is not directly proportional to the expression of IGF-I (53), suggesting that IGFBP-2 may play a crucial role in the hippocampus that is independent of IGF binding.

## Neurotropic and Regenerative Functions OF IGFBP-2 IN CNS

**Figure 3:**
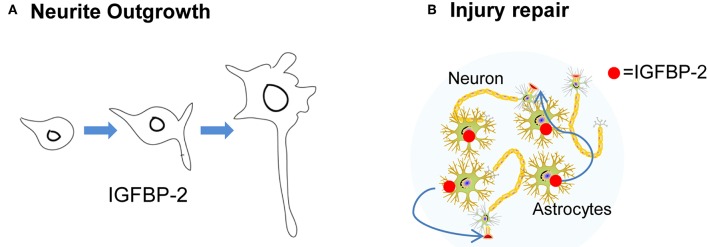
The neurotropic function of IGFBP-2. **(A)** IGFBP-2 is involved in neurite outgrowth. **(B)** IGFBP-2 plays a crucial role in CNS injury and repair.

It is been reported that a variety of neurological insults can enhance IGFBP-2 expression. For example, cerebral hypoxic-ischemia in the neonatal rat results in a loss of IGFBP-2 and IGF-IR expression. In addition, prolonged hypoxia decreased the expression of IGFBP-2, whereas shorter hypoxia insult showed an increase above control. In another study, prolonged hypoxia led to enhanced expression of IGFBP-2 with extensive neuronal loss in the ligated hemisphere as compared to the control hemisphere. Therefore, hypoxia leads to alterations in IGFBP-2 expression (41, 64). Different patterns of IGFBP-2 expression associated with different degrees of injury suggest that IGFBP-2 may contribute to neuronal rescue and/or brain repair processes [Figure 3; (65)]. Similarly, cryogenic spinal cord injury in the rat causes an increase in IGFBP-2 and IGF-IR expression in oligodendrocytes (66). IGFBP-2 and IGF-II expression were enhanced but IGF-IIR expression was decreased after cytotoxic lesion of the rat dentate gyrus (DG). Likewise, a marked increase of IGFBP-2 is observed in astrocytes, neurons, and monocytic cells after brain injury (67). Another rat contusion model showed an increase in both IGFBP-2 and IGF-I adjacent to the injured area (68). The same phenomenon is also observed in perineuronal astrocytes following facial nerve transaction (69). In the case of cryogenic spinal cord injury, at 3 days IGF-I mRNA expression was detected. After 14 days of the lesion, both IGF-I and IGFBP-2 expression was detected, suggesting that both of these peptides have independent and cooperative functions at different stage of repair process. At 28 days after lesion, IGF-IR was detected indicating its role at the later stage of repair process. Thus, the nature of interaction between IGFBP-2/GF-IR may be the “fine-tuning” of healing (66). In another study of cerebral wounds, IGF-I, IGFBP-2, and IGF-IR were detected at 1–7 days post-lesion, suggesting a “cooperative nature” of interaction may exist between IGFBP-2 and IGF-IR (67). In hypoxic-ischemic injury model, IGFBP-2 levels significantly enhanced after 2 days but no noticeable change observed in IGF-IR levels, suggesting an independent of IGFBP-2 in repair process (68). Thus, the nature of IGFBP-2 functions and interaction depend on the type of injury. Together, these findings suggest that IGFBP-2 interaction with IGF-IR may participate in neuronal regeneration in the CNS depending on the nature of injury.

It has been reported that transgenic mice overexpressing IGFBP-2 lacking a specific heparin-binding domain (HBD-1) showed severe deficits in brain growth throughout their lifetime. In addition, these mice when young (12–21 days) had reduced levels of GTPase dynamin-I, and 12 weeks old mice showed weight reduction in the hippocampus, prefrontal cortex, cerebellum, and olfactory bulb, concomitant with reduced myelin basic protein in the cerebellum (17). These data imply that IGFBP-2 is not only involved in brain development but also with information processing and cognition. In addition to supporting neuronal regeneration, IGFBP-2 actions include the ability to promote neurite outgrowth via IGF-IR (70). Shen et al. reported that IGFBP-2 enhances neural stem cells (NSCs) proliferation and maintenance. Moreover, knockdown of IGFBP-2 significantly reduced the expression of cell cycle, differentiation, Notch pathway genes in NSCs (71).

It was found that mice with chronic immobilization stress-induced depressive-like behavior showed reduced expression of IGFBP-2 in the central amygdala (70) and prenatal stress resulted in decreased expression of IGFBP-2 in the hippocampus and frontal cortex in adult male rats (72). When relatively higher concentrations of IGFBP-2 were administered with IGF-II, there was an increased percentage of neurite-bearing cells and an increased average of neurite length (70). Such neurite outgrowth is mediated by IGF-IR associated signaling. During hippocampal neurogenesis at the embryonic stage, there was a dynamic change of gene expression in response to leaf extract of Ginkgo biloba (73). The mRNA level of IGFBP-2 was increased in the fetal hippocampus by prenatal exposure to Ginkgo biloba extract, suggesting that IGFBP-2 may be involved in brain development by acting as both a survival and a differentiation factor for the neural cells (73). High expression of IGFBP-2 in the DG indicates that this protein may be involved in hippocampal neurogenesis. Evidence also suggests that the expression of IGFBP-2 may enhance regenerative sprouting and contribute to neuronal repair in a sensory spinal axonal injury model in the rat (74). On the other hand, mitochondrial dysfunction in Alzheimer's disease (AD) may be associated with enhanced expression of IGFBP-2 (75). AD patients have elevated levels of plasma and CSF IGFBP-2 compare to normal old individuals and plasma IGFBP-2 associated with AD-like brain atrophy (76, 77). Moreover, blood protein analysis showed that IGFBP-2 levels enhanced in serum before the onset of clinical features of AD (78). However, there is a relation between IGFBP-2 and CSF Aβ-42 was observed in the hippocampus. Interestingly, smaller hippocampal volumes associated with higher IGFBP-2 levels only in the amyloid negative individuals (79). Thus, in an age-dependent manner, IGFBP-2 may differentially modulate normal physiological and pathological functions (6, 80).

The ability of IGFBP-2 to promote neuronal survival is associated with its ability to prevent apoptosis. The Bcl-2 (B-cell lymphoma 2) family of proteins is important in the regulation of apoptosis, and BCL-2 mRNA is localized in the hippocampus at the same sites of IGF/IGFBPs expression (81). The IGF/IGFBPs system and the pro-survival Bcl-2 proteins protect cells from apoptosis and play a key role in brain development. Moreover, IGFBP-2 expression is markedly increased around the site of injury (82), thus providing evidence that IGFBP-2 may inhibit neuronal apoptosis. Baker et al. (83) showed that IGFBP-2 exerts anti-apoptotic effects in developing mouse brain probably via Bcl-2 overexpression. This resulted in the induction and overexpression of IGFBP-2 in mitral cells, demonstrating a previously unknown mechanism for cell survival by Bcl-2 via IGFBP-2 in developing brain (83). On the other hand, IGFBP-2 may have some link with psychiatric disorders. For example, serum IGFBP-2 level was significantly higher in schizophrenic patients than control (84, 85). Serum IGFBP-2-protein and -mRNA expression was downregulated in patients with bipolar disease (86, 87), and serum IGFBP-2 protein levels were lower in patients with atypical and melancholic depression than in controls (88). Moreover, it has been reported that the brains of individuals with schizophrenia showed faster aging than normal (89). Mitochondrial dysfunction plays a key role in neuropsychiatric disorders including schizophrenia (90). Thus, senescent cells may have mitochondrial dysfunction and elevated IGFBP-2. On the other hand, fibrotic lung disease is characterized by the presence of senescent cells, where IGFBP-2 expression was moderately high (91). Stress-induced cellular senescence often recognized by the expression of p16Ink4a accumulates in various tissues. Senescence positive cells showed higher expression of IGFBP-2 (92). Thus, IGFBP-2 may associate with cellular senescence related to psychiatric disorders.

The number of IGFBP-2-positive cells was decreased in the hippocampus of a mouse model of amyotrophic lateral sclerosis (ALS) (93). IGF-l is a growth facilitator that propels axon regeneration and can restore corticospinal axon function (94). Chromatin remodeling via histone methylation is critical for peripheral nerve myelination by Schwann cells. Knock-out of Schwann cell-specific subunit of the polycomb repressive complex-2 (PRC-2) called Eed, which catalyzes methylation of histone H3 Lys27 causes hypermyelination of axon. In addition, such knock out of Eed was responsible for enhanced Akt phosphorylation accompanied by decreased expression of *IGFBP2* gene (95). Thus, these data in addition to down-regulation of IGFBP-2 in the distal stump of sciatic nerve cut model mice (c-Jun deleted in Schwann cells) (96), implying that IGFBP-2 is a potential factor in the epigenetics pathway for mature myelinated axons.

## Bidirectional Effect Of IGFBP-2 On CNS Myelination

**Figure 4:**
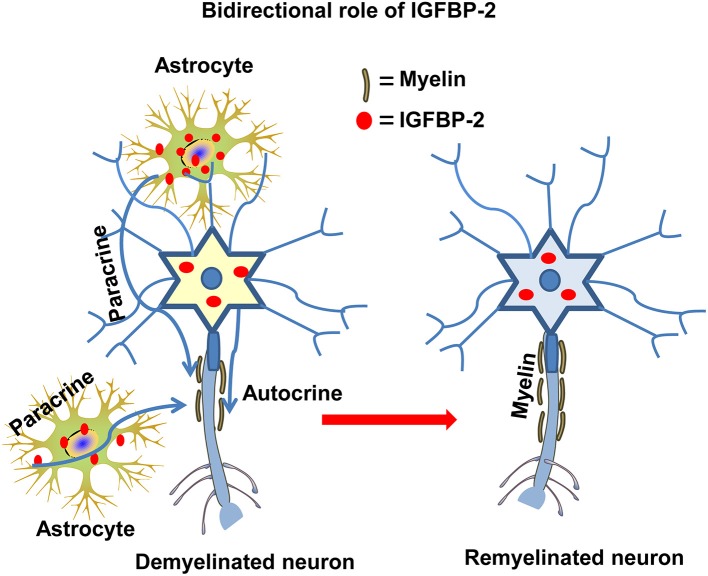
The autocrine and paracrine action of IGFBP-2 in myelination. IGFBP-2 is involved in myelination and myelin repair via autocrine and paracrine mechanisms both in CNS and PNS neurons.

It has been reported that IGF-I increases myelination, while IGFBP-1 suppresses myelination (97). Immunoreactivity of IGF-II and IGFBP-2 colocalized on oligodendrocyte membrane and myelin sheaths (98). It may possible that IGFBP-2 is transported on myelin tracks from its sites of synthesis. In another study, IGFBP-2 and IGF-II colocalized in sites remote from their expression sites, especially in the myelin sheaths of axons and nerve tracts in the brain [Figure 4; (99)]. This spatial discrepancy between the sites of synthesis and the sites of localization suggests a bidirectional role of IGFBP-2: transport of IGF-II to its target cells and IGFBP-2's own independent functions. In addition, during encephalomyelitis, IGFBP-2 expression in reactive astrocytes targets oligodendrocytes, expressing IGF-I and IGF-IR, which are responsible for remyelination (100). In contrast, *in vitro* treatment of primary astrocytes with IGF-I/IGFBP-2 complexes revealed no inhibitory effect on IGF-I induced proliferation, but the same complexes significantly inhibit IGF-I induced survival of oligodendrocytes (101). In addition, peripheral nerve myelination is mediated by IGFBP-2 via Akt activation in Schwann cells (95, 102). Thus, IGFBP-2 may follow differential mechanisms for central and peripheral myelination.

Interestingly, gray matter astrocytes express high levels of IGFBP-2, whereas IGF-I mRNA and IGF-I peptide are not detected. This dissociated astrocytic expression suggests that IGFBP-2 and IGF-I responses may be regulated differentially by neurons. In cryogenic spinal cord injury, it has been reported that IGFBP-2 expression was significantly increased by astrocytes (66), suggesting that IGFBP-2 may have a role in promoting myelin regeneration. However, oligodendrocytes treated with IGFBP-2 revealed decreased expression of myelin protein and myelin-associated glycoprotein (103). *In vitro*, IGFBP-2 attenuated cell differentiation by reducing IGF-I. In contrast, cryogenic spinal cord injury leading to demyelination and axonal loss resulted in enhanced IGF-I and IGFBP-2 production by astrocytes (104). In another study in the human adult brain, IGFBP-1-6 has been shown in astrocytes but not in microglia (101, 105). Chronic active multiple sclerosis (MS) lesions showed enhanced expression of IGFBP-2 and IGFBP-4 in astrocytes but not in microglia or macrophages. Although the signaling pathway for enhanced expression of IGFBP-2 in MS remains unknown, up-regulation of IGF-I and IGF-II, including proinflammatory cytokines, may be responsible for enhanced IGFBP-2 expression in MS.

Moreover, cytokines and epidermal growth factor (EGF) can induce specific IGFBPs production which plays a role in the cell growth in human salivary cell line (HSG) (106). Thus, Cytokines include IL-1b (interleukin-1b), IL-6 (interleukin-6); TNF-a (Tumor necrosis factor-alpha), and IFN-g (interferon-gamma), all of which are proinflammatory cytokines (hallmark of persistent inflammation), responsible for enhanced expression of IGFBP-2 in astrocytes and microglia and, a feature of active MS (18, 107). Thus, it was hypothesized that IGFBP-2 may protect cells from cytokine release, an effect that is mediated by IGFs and which may enhance astrogliosis (18). In addition, the induction of encephalomyelitis in rats resulted in enhanced expression of IGFBP-2 and IGF-I in astrocytes and coincided with remyelination by targeting promyelinating oligodendrocytes (106). Thus, it is assumed that IGFBP-2 may be a therapeutic target for MS treatment via mechanisms where IGFBP-2 differentially modulates CNS myelination during pathological and normal physiological conditions.

## Possible Functions Of IGFBP-2 In The Hippocampus

The components of the IGF system are highly expressed in regions that are undergoing remodeling or enhanced plasticity, such as the hippocampus. It was demonstrated that IGF-IR colocalized to or near sites of IGF/IGFBPs expression in the hippocampus. Moreover, IGFBP-2 binds to cell surface proteoglycans (39). It is suggested, therefore, that there is an autocrine or paracrine mode of IGFBP-2 action in the developing hippocampus (108). Cell type specific expression and distribution of IGFBP-2 suggest complex regulation of IGFBP-2 action in the CNS.

IGF-IR is expressed at high levels during development by most regions of the CNS (1, 109). However, IGF-IR shows significantly different structural characteristics in non-neural tissues from those expressed by the brain. The major difference is that the carbohydrate residues of the peripheral IGF-IR include N-linked high-mannose and contain sialic acid molecules. On the other hand, in the neural IGF-IR, a polymer of sialic acid is present and is resistant to neuraminidase catalysis (110, 111). In some cases, sialic acid residues may be absent in neuronal IGF-lR. Such alterations may be responsible for the different affinities of the ligands with neuronal IGF-IR, which may explain the differential roles of the IGF/IGFBP complex in the brain. Interestingly, glial cells express the non-neuronal type IGF-IR (109, 112), suggesting that neurons are specialized for functions that are different from those of glia.

In developing the hippocampus, IGF-IR mRNA is abundant in pyramidal neurons (PNs), granule cells, and interneurons (111). Moreover, PNs show high levels of IGF-IR gene expression in conjunction with local IGFBP-2 expression (53, 70, 113, 114), demonstrating that IGFBP-2 may act via IGF-IR in the hippocampus. There is relatively stable and uniform level of IGF-IR gene expression in all neuroepithelial lineages, indicating this pattern of receptor distribution may be the target for peripheral IGF/IGFBPs and subserve a very basic metabolic or trophic function, demonstrating that there may be specific local autocrine and/or paracrine IGF/IGFBPs actions mediated by IGF-IR in the brain (108). IGFBP-2 has been reported to prolong and modulate cellular functions independent of IGF-I binding (3), and IGFBP-2 can prevent IGF-I from binding to its receptor (20, 114). Therefore, IGF-IR activation may be required for IGFBP-2 functions. The C-tail region of IGF-IR is the site for binding of different signaling molecules. For IGFBPs binding to IGF-IR, a single detergent molecule contacts residues known to be critical (111). Proteolytic degradation of IGFBP-2 near cell surface receptors has lower affinity for IGFs and thus by competing with IGFs, HBD of IGFBP-2 may bind with that detergent molecule in IGF-IR to initiate downstream signaling via autophosphorylation of the receptor (41). Moreover, the presence or absence of sialic acid residue in the IGF-IR may be another factor for interaction with IGFBP-2.

Moreover, Khan et al. (41) showed that the HBD domain of IGFBP-2 can influence AMPA and GABA receptors reflected by enhanced miniature EPSC and IPSC frequency and amplitudes, while treatment with IGF-IR antagonist (JB-1) significantly reduced those events, suggesting that HBD domain may interact with the IGF-IR. Interestingly, microinjection of IGFBP-2 in wild type (WT) and *igfbp2*^−/−^ mice showed no change in IGF-I/II levels, indicating no needs IGFs for HBD domain of IGFBP-2 to bind with the receptors (41). It may also be possible that the interaction between IGFBP-2 and IGF-IR may induce a rapid and transient increase in intracellular free calcium concentration (115), which is responsible for enhanced synaptic plasticity. Moreover, the bath application of HBD domain of IGFBP-2 significantly enhanced and rescued long term potentiation (LTP) in WT and *igfbp2*^−/−^ mice, respectively. In spatial learning and memory test, *igfbp2*^−/−^ mice showed slower learning profile and spent less time in the target quadrate (41). Thus, IGFBP-2 may differentially modulate both the short-term and long-term synaptic plasticity.

In the HBD domain, IGFBP-2 possesses nuclear localization signal (NLS) motif (116) which performs various non-IGF-IR dependent functions such as IGFBP-2 (HBD domain) involves in bone formation via both IGF-dependent and IGF-independent mechanisms (117–119). Moreover, HDB domain of IGFBP-2 can rescue differentiation and the expression of osteocalcin in *igfbp2*^−/−^ mice via RPTPβ (120). Moreover, IGFBP-2 inhibits adipogenesis and fat development without interacting with IGFs (121). In addition, IGFBP-2 down-regulates the tumor suppressor gene phosphatase and tensin homolog (PTEN) via an integrin-mediated mechanism to enhance IGF-I induced Akt pathway activation (37). It is worth emphasizing that IGFBP-2 could derive either from neurons or astrocytes (105, 122, 123), because there may be a source independent of local autocrine and/or paracrine action of IGFBP-2 (Figure 4) that is mediated by IGF-IR (102) rather than by the integrin (124).

Electroconvulsive seizure therapy (ECS) is a clinically proven treatment for depression. ECS increases the expression of specific neurotrophic factors that could block or reverse the atrophy and cell loss resulting from stress and depression. Research into the mechanism of action of ECS has reported that expression of neurotrophic-growth factors and related signaling pathways in the hippocampus and in the choroid plexus were enhanced, both areas where IGFBP-2 is highly expressed (125). In adult male rats, IGFBP-2 (1 μg/kg, i.v.) increased the density of mature dendritic spines and total spine density in the DG and medial prefrontal cortex, which is the underlying mechanisms explaining the therapeutic-like effects of IGFBP-2 in post-traumatic stress disorder (PTSD) (126). In most cases, depressive symptoms associated with PTSD (127). Intracerebroventricular (icv) injection of NBI-31772 (10–30 mg; a non-specific IGFBP inhibitor) enhanced anxiolytic-like behavior in four-plate test and elevated zero maze tests. On the contrary, treatment with the same NBI-31772 (3–30 mg) showed decreased immobility time in the tail suspension test, an indicator of antidepressant-like effects (128). In another study, exposure to stressful stimuli showed depression-like behavior in rats accompanied by reduced IGF-l protein levels in the frontal cortex and in the hippocampus with differential expression of IGFBPs. IGFBP-2 and IGFBP-3 levels were decreased while IGFBP-4 was increased with no change in IGFBP-1 and IGFBP-6 in those stressed rats (72). Taken together, these data suggest that IGFBP-2 acts as an antidepressant and anxiolytic agent by reducing stress-induced cell loss or atrophy, which is consistent with the finding that persons with atypical depression showed lower levels of IGFBP-2 (93).

In addition, IGFBP-2 is also involved in neurodegeneration. It has been reported that the accumulation of high levels of Abeta can be toxic, although the alpha-secretase cleaved amyloid precursor protein (APP) is neuroprotective because it increases the expression levels of several neuroprotective genes, including IGFBP-2. Thus, IGFBP-2 may protect hippocampal neurons from Abeta-induced tau phosphorylation and neuronal death in AD (129). This finding is consistent with the notion that knocking out the *IGFBP2* gene may reduce hippocampal cell number. In addition, in age impaired rats, G-protein coupled receptors and other signaling processes were down-regulated in the hippocampus, whereas IGFBP-2 expression was unaltered, suggesting that IGFBP-2 may not be involved in G-protein-coupled receptor signaling (130).

Dynamic synaptic plasticity is critical for learning and memory throughout life. It has already been reported that insulin-like peptide plays an important role in neurite outgrowth (1). Moreover, IGFBP-2 increased total spines number in dentate granule neurons of the hippocampus and mature spine density in medial prefrontal cortex (MPFC) layer-V pyramidal neurons (126). Thus, IGFBP-2 may involve in increasing the number of functional synapses which can be the underlying mechanism of enhanced LTP by IGFBP-2. Interestingly, intermittent hypoxia (IH, 16% O_2_) enhanced spatial learning and memory (41, 131). It was reported that IH enhanced expression of IGFBP-2, IGF-IR, CRF, and CRF-lR in neonatal hippocampus (41, 132). Additionally, CRF enhanced the propagation of neuronal activity from DG to the CA1 region (133). Thus, there may be interplay between IGFBP-2 and CRF leading to enhanced synaptic plasticity (41). Diabetes differentially modulates IGFBP-2 levels in organs (134). In addition, diabetes impairs hippocampal function through glucocorticoid (135), whereas stress (e.g., hypoxia) enhances IGFBP-2 levels in the hippocampus (132, 136). Taken together, this could signal the interplay between IGFBP-2 and stress hormones in the hippocampus in physiological and pathological conditions. In this case, IGFBP-2 may directly modulate neural functions independent of IGF-IR.

## Concluding Remarks And Prospects

**Figure 5:**
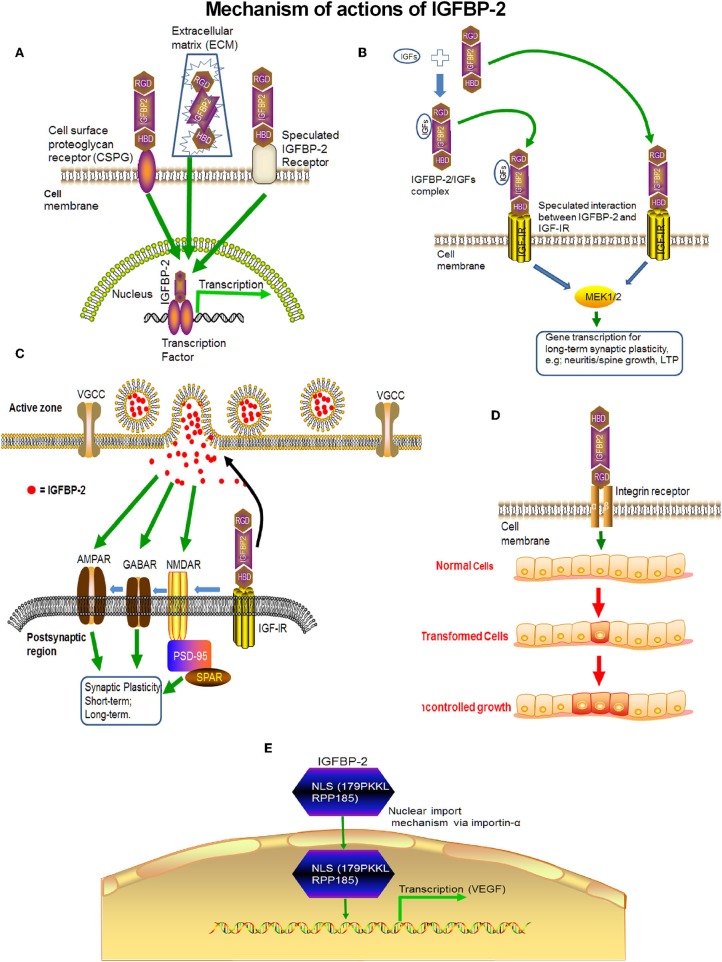
The probable mechanisms of action of IGFBP-2 in the CNS. **(A)** IGFBP-2 may directly bind with putative IGFBP-2 receptors on the neural membrane and may initiate transcription. **(B)** IGFBP-2 may bind with IGF-IR via the HBD domain and may initiate downstream signaling via the ERK1/2-MAPK pathway. **(C)** After binding with IGF-IR via the HBD domain, IGFBP-2 may transfer the signal to AMPAR, GABAR, or NMDAR to modulate neurotransmission for short term synaptic plasticity. **(D)** IGFBP-2 binds with integrin receptors via the RDG-domain and initiates malignancy. **(E)** IGFBP-2 may directly cross the cell membrane via nuclear localization signaling and initiate transcription.

A variety of signaling pathways and extracellular factors such as IGF-I/II, corticotrophin releasing factor (CRF), glucocorticoid, TGF-beta, interleukin 1, and estradiol may influence IGFBP-2 expression (137). In addition, several physiological conditions such as fasting, stroke, and hypoxia enhanced IGFBP-2 levels and IGFBP-2 locally expressed in the CNS. Thus, there is a complex regulation and mechanism of action of IGFBP-2 in the CNS. IGFBP-2 may act by itself or via with IGF-IR or indirectly via IGF-I and/or IGF-II. As a consequence, IGFBP-2 seems to have four or more mechanisms (Figure 5) of action in the CNS by which it mediates its own independent functions: (1) The binding of the IGFBP-2 via the HBD domain to putative receptors on the cell membrane may stimulate the signaling pathway independent of IGF-R and mediate the effect of IGFBP-2 in a cell-type-specific manner. (2) Since IGFBP-2 co-localized with IGF-IR in the hypothalamus (138), thus IGFBP2 may initiate downstream signaling (1). (3) IGFBP-2 may transport into the nucleus via its nuclear localization signal and cause transcriptional activation of genes (116). (4) IGFBP-2 can bind with the integrin receptor via the RGD domain, which happens in cases of CNS malignancy but not in the normal physiological function of neurons. Different mechanisms are crucial for the different physiological functions of IGFBP-2. For example, binding to a neural membrane receptor (e.g., IGF-IR, AMPAR, GABAR, and NMDAR) via the HBD domain may be responsible for neurotransmission and synaptic plasticity, whereas direct transport to the nucleus may be responsible for neurite outgrowth. In addition, it may also be possible that these mechanisms of IGFBP-2 function may overlap with each other and their interplay may be responsible for the conversion of short-term plasticity to long-term memory. IGFBP-2, therefore, exhibits spatial and temporal patterns of action.

**Figure 6:**
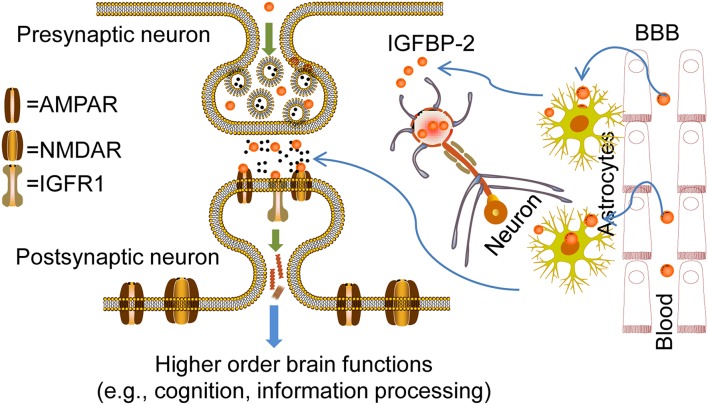
Overview of IGFBP-2 action in CNS for higher order brain functions. Probable mechanisms for the entry of IGFBP-2 across the blood-brain barrier (BBB) to cerebrospinal fluid (CSF): Transport could happen via transcytosis through the choroid plexus where low-density lipoprotein receptor-related proteins-1/2 (LRP-1/2) and IGF-IR may play a crucial role in IGFBP-2 entry from blood to CSF. In this type of entry, concentrations of IGFBP-2 in the blood determine entry. IGFBP-2 in the CSF interacts with IGF-IR in the hippocampus because of its location adjacent to the ventricular system. IGFBP-2 released from astrocytes in response to any kind of CNS insult may directly interact with IGF-IR and be involved in the CNS repair process, neural development, and growth. On the other hand, IGFBP-2 in the neurons is released upon neuronal excitability. In this case, IGFBP-2 may directly bind with the receptors such as AMPAR, GABAR, and NMDAR in the dendrites and axon terminals to mediate synaptic plasticity mechanisms for higher-order brain function, from information processing to cognition. In addition, IGFBP-2 may phosphorylate different ion channels by phosphoinositide 3-kinase (PI3K) or extracellular signal-related kinases (ERKs). IGFBP-2 synthesized inside the neurons may regulate the synthesis of synaptic proteins by direct localization into the nucleus.

IGFBP-2 expression in hippocampal neurons and astrocytes peaks during brain development, coincidently when neuron progenitor proliferation and/or neuritic outgrowth occur, while expression of IGF-IR appears ubiquitous. This expression pattern of IGFBP-2 during brain growth suggests highly regulated and developmentally timed IGFBP-2 actions on specific neural cell populations. Several studies clearly implicate IGFBP-2 involvement in specific higher-order brain functions via spatial and temporal regulations. Thus, IGFBP-2 is not only a binding protein but a crucial CNS growth factor that is responsible for cognition and information processing in the brain (Figure 6). Although targeting of IGFBP-2 signaling to particular cell types remains to be established, IGFBP-2 not only regulates brain development at early embryonic stages but is also crucial in neuronal and adult stages for higher-order brain functions, such as learning, memory, and information processing. IGFBP-2 is revealing its many secrets not only in the embryonic stage but also in other multi-faceted areas from neuroprotection to cognitive function, thus it spans from bench to bedside.

## Author Contributions

The author confirms being the sole contributor of this work and has approved it for publication.

### Conflict of Interest

The author declares that the research was conducted in the absence of any commercial or financial relationships that could be construed as a potential conflict of interest.

## Abbreviations

IGFBPs: insulin-like growth factor binding proteins
IGFBP-2: insulin-like growth factor binding protein-2
CSF: cerebrospinal fluid
IGFs: insulin-like growth factors
IGF-I: insulin-like growth factor-1
IGF-II: insulin-like growth factor-2
HBD: heparin binding domain
IGF-IR: insulin-like growth factor receptor-1
IGF-IR: insulin-like growth factor receptor-2
CNS: central nervous system
RGD domain: Arginine-Glycine-Aspartate tripeptide
DG: dentate gyrus
MS: multiple sclerosis
ECM: extracellular matrix
GTPase: guanosine triphosphatase
BBB: blood brain barrier
mRNA: messenger ribonucleic acid
AD: Alzheimer's disease
ALS: amyotrophic lateral sclerosis
Bcl-2: B-cell lymphoma 2
Akt: serine/threonine-specific protein kinase
IL: interleukin
TNF-a: tumor necrosis factor alpha
IFN-g: interferon gamma
AMPAR: α-amino-3-hydroxy-5-methyl-4-isoxazolepropionic acid receptor
GABAR: gamma-aminobutyric acid receptor
NMDAR: N-methyl-D-aspartate receptor
ECS: electroconvulsive seizure therapy
APP: amyloid precursor protein
LTP: long term potentiation
CRF: corticotrophin releasing factor
CRF-lR: corticotrophin releasing factor receptor 1
IH: intermittent hypoxia
CA1: Cornu Ammonis-1
PNs: pyramidal neurons.

## References

[1] Fernandez AM Torres AI. The many faces of insulin-like peptide signalling in the brain. Nat Rev Neurosci. (2012) 13:225–39. 10.1038/nrn3209 22430016

[2] Yu H Rohan T. Role of the insulin-like growth factor family in cancer development and progression. J Natl Cancer Inst. (2000) 92:1472–89. 10.1093/jnci/92.18.1472 10995803

[3] Hoeflich A Reisinger R Lahm H Kiess W Blum WF Kolb HJ Insulin-like growth factor-binding protein 2 in tumorigenesis: protector or promoter? Cancer Res. (2001) 61:8601–10. 11751371

[4] Bianchi VE Locatelli V Rizzi L. Neurotrophic and neuroregenerative effects of GH/IGF1. Int J Mol Sci. (2017) 18:E2441. 10.3390/ijms18112441 29149058 PMC5713408

[5] Dyer AH Vahdatpour C Sanfeliu A Tropea D. The role of insulin-like growth factor 1 (IGF-1) in brain development, maturation and neuroplasticity. Neuroscience. (2016) 325:89–99. 10.1016/j.neuroscience.2016.03.056 27038749

[6] Ziegler AN Levison SW Wood TL. Insulin and IGF receptor signalling in neural-stem-cell homeostasis. Nat Rev Endocrinol. (2015) 11:161–70. 10.1038/nrendo.2014.208 25445849 PMC5513669

[7] Chen DY Stern SA Garcia OA Saunier RB Pollonini G Bambah MD. A critical role for IGF-II in memory consolidation and enhancement. Nature. (2011) 469:491–7. 10.1038/nature09667 21270887 PMC3908455

[8] Mardinly AR Spiegel I Patrizi A Centofante E Bazinet JE Tzeng CP. Sensory experience regulates cortical inhibition by inducing IGF1 in VIP neurons. Nature. (2016) 531:371–5. 10.1038/nature17187 26958833 PMC4823817

[9] Shcheglovitov A Shcheglovitova O Yazawa M Portmann T Shu R Sebastiano V. SHANK3 and IGF1 restore synaptic deficits in neurons from 22q13 deletion syndrome patients. Nature. (2013) 503:267–71. 10.1038/nature12618 24132240 PMC5559273

[10] Mozell RL McMorris FA. Insulin-like growth factor I stimulates oligodendrocyte development and myelination in rat brain aggregate cultures. J Neurosci Res. (1991) 30:382–90. 10.1002/jnr.490300214 1665869

[11] Recio PE Lang FF Ishii DN Insulin and insulin-like growth factor II permit nerve growth factor binding and the neurite formation response in cultured human neuroblastoma cells. Proc Natl Acad Sci USA. (1984) 81:2562–6. 10.1073/pnas.81.8.2562 6326132 PMC345103

[12] Kimpinski K Mearow K. Neurite growth promotion by nerve growth factor and insulin-like growth factor-1 in cultured adult sensory neurons: role of phosphoinositide 3-kinase and mitogen activated protein kinase. J Neurosci Res. (2001) 63:486–99. 10.1002/jnr.1043 11241584

[13] Mason JL Xuan S Dragatsis I Efstratiadis A Goldman JE. Insulin-like growth factor (IGF) signaling through type 1 IGF receptor plays an important role in remyelination. J Neurosci. (2003) 23:7710–8. 10.1523/JNEUROSCI.23-20-07710.2003 12930811 PMC6740767

[14] Werner H Adamo M Roberts CT LeRoith D. Molecular and cellular aspects of insulin-like growth factor action. Vitam Horm. (1994) 48;1–58. 10.1016/S0083-6729(08)60495-1 7524243

[15] Sehat B Tofigh A Lin Y Trocmé E Liljedahl U Lagergren J. SUMOylation mediates the nuclear translocation and signaling of the IGF-1 receptor. Sci Signal. (2010) 3:ra10. 10.1126/scisignal.2000628 20145208

[16] Hawkes C Jhamandas JH Harris KH Fu W MacDonald RG Kar S. Single transmembrane domain insulin-like growth factor-II/mannose-6-phosphate receptor regulates central cholinergic function by activating a G-protein-sensitive, protein kinase C-dependent pathway. J Neurosci. (2006) 26:585–96. 10.1523/JNEUROSCI.2730-05.2006 16407557 PMC6674423

[17] Schindler N Mayer J Saenger S Gimsa U Walz C Brenmoehl J. Phenotype analysis of male transgenic mice overexpressing mutant IGFBP-2 lacking the Cardin-Weintraub sequence motif: reduced expression of synaptic markers and myelin basic protein in the brain and a lower degree of anxiety-like behaviour. Growth Horm IGF Res. (2017) 33:1–8. 10.1016/j.ghir.2016.11.003 27919008

[18] Chesik D De KJ Wilczak N. Insulin-like growth factor binding protein-2 as a regulator of IGF actions in CNS: implications in multiple sclerosis. Cytokine Growth Factor Rev. (2007) 18:267–78. 10.1016/j.cytogfr.2007.04.001 17485236

[19] Ocrant I Fay CT Parmelee JT. Characterization of insulin-like growth factor binding proteins produced in the rat central nervous system. Endocrinology. (1990) 127:1260–7. 10.1210/endo-127-3-1260 1696881

[20] Firth SM Baxter RC. Cellular actions of the insulin-like growth factor binding proteins. Endocr Rev. (2002) 23:824–54. 10.1210/er.2001-0033 12466191

[21] Galea CA Mobli M McNeil KA Mulhern TD Wallace JC King GF. Insulin-like growth factor binding protein-2: NMR analysis and structural characterization of the N-terminal domain. Biochimie. (2012) 94:608–16. 10.1016/j.biochi.2011.09.012 21951978

[22] Russo VC Rekaris G Baker NL Bach LA Werther GA. Basic fibroblast growth factor induces proteolysis of secreted and cell membrane-associated insulin-like growth factor binding protein-2 in human neuroblastoma cells. Endocrinology. (1999) 140:3082–90. 10.1210/endo.140.7.6771 10385400

[23] Hobba GD Forbes BE Parkinson EJ Francis GL Wallace JC. The insulin-like growth factor (IGF) binding site of bovine insulin-like growth factor binding protein-2 (bIGFBP-2) probed by iodination. J Biol Chem. (1996) 271:30529–36. 10.1074/jbc.271.48.30529 8940022

[24] Carrick FE Hinds MG McNeil KA Wallace JC Forbes BE Norton RS. Interaction of insulin-like growth factor (IGF)-I and -II with IGF binding protein-2: mapping the binding surfaces by nuclear magnetic resonance. J Mol Endocrinol. (2005) 34:685–98. 10.1677/jme.1.01756 15956340

[25] Arai T Busby W Jr Clemmons DR. Binding of insulin-like growth factor (IGF) I or II to IGF-binding protein-2 enables it to bind to heparin and extracellular matrix. Endocrinology. (1996) 137:4571–5. 10.1210/endo.137.11.8895319 8895319

[26] Chelsky D Ralph R Jonak G. Sequence requirements for synthetic peptide-mediated translocation to the nucleus. Mol Cell Biol. (1989) 9:2487–92. 10.1128/MCB.9.6.2487 2668735 PMC362321

[27] Fowlkes JL Serra DM. Characterization of glycosaminoglycan-binding domains present in insulin-like growth factor-binding protein-3. J Biol Chem. (1996) 271:14676–9. 10.1074/jbc.271.25.14676 8663298

[28] Jones JI Gockerman A Busby WH Jr Wright G Clemmons DR. Insulin-like growth factor binding protein 1 stimulates cell migration and binds to the alpha 5 beta 1 integrin by means of its Arg-Gly-Asp sequence. Proc Natl Acad Sci USA. (1993) 90:10553–7. 10.1073/pnas.90.22.10553 7504269 PMC47815

[29] Hoeflich A Reisinger R Vargas GA Elmlinger MW Schuett B Jehle PM Mutation of the RGD sequence does not affect plasma membrane association and growth inhibitory effects of elevated IGFBP-2 in vivo. FEBS Lett. (2002) 523:63–7. 10.1016/S0014-5793(02)02935-6 12123805

[30] Daza DO Sundstrom G Bergqvist CA Duan C Larhammar D. Evolution of the insulin-like growth factor binding protein (IGFBP) family. Endocrinology. (2011) 152:2278–89. 10.1210/en.2011-0047 21505050

[31] Wang GK Hu L Fuller GN Zhang W. An interaction between insulin-like growth factor-binding protein 2 (IGFBP2) and integrin alpha5 is essential for IGFBP2-induced cell mobility. J Biol Chem. (2006) 281:14085–91. 10.1074/jbc.M513686200 16569642

[32] Song SW Fuller GN Khan A Kong S Shen W Taylor E. IIp45, an insulin-like growth factor binding protein 2 (IGFBP-2) binding protein, antagonizes IGFBP-2 stimulation of glioma cell invasion. Proc Natl Acad Sci USA. (2003) 100:13970–5. 10.1073/pnas.2332186100 14617774 PMC283530

[33] Frommer KW Reichenmiller K Schutt BS Hoeflich A Ranke MB Dodt G. IGF-independent effects of IGFBP-2 on the human breast cancer cell line Hs578T. J Mol Endocrino. (2006) 37:13–23. 10.1677/jme.1.01955 16901920

[34] Pereira JJ Meyer T Docherty SE Reid HH Marshall J. Thompson EW. Bimolecular interaction of insulin-like growth factor (IGF) binding protein-2 with alphavbeta3 negatively modulates IGF-I-mediated migration and tumor growth. Cancer Res. (2004) 64:977–84. 10.1158/0008-5472.CAN-03-3056 14871828

[35] Perks CM Vernon EG Rosendahl AH Tonge D Holly JM. IGF-II and IGFBP-2 differentially regulate PTEN in human breast cancer cells. Oncogene. (2007) 26:5966–72. 10.1038/sj.onc.1210397 17369847

[36] Russo VC Schutt BS Andaloro E Ymer SI Hoeflich A Ranke MB. Insulin-like growth factor binding protein-2 binding to extracellular matrix plays a critical role in neuroblastoma cell proliferation, migration, and invasion. Endocrinology. (2005) 146:4445–55. 10.1210/en.2005-0467 15994346

[37] Shen X Xi G Maile LA Wai C Rosen CJ Clemmons DR. Insulin-like growth factor (IGF) binding protein 2 functions coordinately with receptor protein tyrosine phosphatase and the IGF-I receptor to regulate IGF-I-stimulated signaling. Mol Cell Biol. (2012) 32:4116–30. 10.1128/MCB.01011-12 22869525 PMC3457336

[38] Kuang Z Yao S Keizer DW Wang CC Bach LA Forbes BE. Structure, dynamics and heparin binding of the C-terminal domain of insulin-like growth factor-binding protein-2 (IGFBP-2). J Mol Biol. (2006) 364:690–704. 10.1016/j.jmb.2006.09.006 17020769

[39] Russo VC Azar WJ Yau SW Sabin MA Werther GA. IGFBP-2: the dark horse in metabolism and cancer. Cytokine Growth Factor Rev. (2015) 26:329–46. 10.1016/j.cytogfr.2014.12.001 25544066

[40] Collett SPF Cohen P The role of the insulin-like growth factor binding proteins and the IGFBP proteases in modulating IGF action. Endocrinol Metab Clin North Am. (1996) 25:591–614. 10.1016/S0889-8529(05)70342-X 8879988

[41] Khan S Lu X Huang Q Tang J Weng J Yang Z IGFBP2 plays an essential role in cognitive development during early life. Adv Sci. (2019). 10.1002/advs.201901152. [Epub ahead of print]. PMC6891907 31832311

[42] Hoeflich A Reyer A Ohde D Schindler N Brenmoehl J Spitschak M. Dissociation of somatic growth, time of sexual maturity, and life expectancy by overexpression of an RGD-deficient IGFBP-2 variant in female transgenic mice. Aging Cell. (2016) 15:111–7. 10.1111/acel.12413 26507795 PMC4717279

[43] Inoue T Hashimoto R Matsumoto A Jahan E Rafiq AM Udagawa J. *In vivo* analysis of Arg-Gly-Asp sequence/integrin α5β1-mediated signal involvement in embryonic enchondral ossification by exo utero development system. J Bone Miner Res. (2014) 29:1554–63. 10.1002/jbmr.2166 24375788

[44] Liu Y Li F Yang YT Xu XD Chen JS Chen TL. IGFBP2 promotes vasculogenic mimicry formation via regulating CD144 and MMP2 expression in glioma.Oncogene. (2018) 38:1815–31. 10.1038/s41388-018-0525-4 30368528

[45] Hoeflich A Wu M Mohan S Foll J Wanke R Froehlich T el al. Overexpression of insulin-like growth factor-binding protein-2 in transgenic mice reduces postnatal body weight gain. Endocrinology. (1999) 140:5488–96. 10.1210/endo.140.12.7169 10579311

[46] Wood TL Rogler LE Czick ME Schuller AG Pintar JE. Selective alterations in organ sizes in mice with a targeted disruption of the insulin-like growth factor binding protein-2 gene. Mol Endocrinol. (2000) 14:1472–82. 10.1210/mend.14.9.0517 10976924

[47] Russo VC Bach LA Werther GA. Cell membrane association of insulin-like growth factor binding protein-2 (IGFBP-2) in the rat brain olfactory bulb. Prog Growth Factor Res. (1995) 6:329–36. 10.1016/0955-2235(95)00018-6 8817676

[48] Conover CA Khosla S. Role of extracellular matrix in insulin-like growth factor (IGF) binding protein-2 regulation of IGF-II action in normal human osteoblasts. Growth Horm IGF Res. (2003) 13:328–35. 10.1016/S1096-6374(03)00092-3 14624766

[49] Russo VC Gluckman PD Feldman EL Werther GA. The insulin-like growth factor system and its pleiotropic functions in brain. Endocr Rev. (2005) 26:916–43. 10.1210/er.2004-0024 16131630

[50] Allard JB Duan C. IGF-binding proteins: why do they exist and why are there so many? Front Endocrinol. (2018) 9:117. 10.3389/fendo.2018.00117 29686648 PMC5900387

[51] Oeflich A Reisinger R Schuett BS Elmlinger MW Russo VC Vargas GA Peri/nuclear localization of intact insulin-like growth factor binding protein-2 and a distinct carboxyl-terminal IGFBP-2 fragment in vivo. Biochem Biophys Res Commun. (2004) 324:705–10. 10.1016/j.bbrc.2004.09.111 15474485

[52] Graham ME Kilby DM Firth SM Robinson PJ Baxter RC. The *In vivo* phosphorylation and glycosylation of human insulin-like growth factor-binding protein-5. Mol Cell Proteomics. (2007) 6:1392–405. 10.1074/mcp.M700027-MCP200 17496250

[53] Lee WH Michels KM Bondy CA. Localization of insulin-like growth factor binding protein-2 messenger RNA during postnatal brain development: correlation with insulin-like growth factors I and II. Neuroscience. (1993) 53:251–65. 10.1016/0306-4522(93)90303-W 7682300

[54] Wood TL Brown AL Rechler MM Pintar JE. The expression pattern of an insulin-like growth factor (IGF)-binding protein gene is distinct from IGF-II in the midgestational rat embryo. Mol Endocrinol. (1990) 4:1257–63. 10.1210/mend-4-8-1257 1705658

[55] Lee WH Javedan S Bondy CA. Coordinate expression of insulin-like growth factor system components by neurons and neuroglia during retinal and cerebellar development. J Neurosci. (1992) 12:4737–44. 10.1523/JNEUROSCI.12-12-04737.1992 1281494 PMC6575757

[56] Tseng LY Brown AL Yang YW Romanus JA Orlowski CC Taylor T. The fetal rat binding protein for insulin-like growth factors is expressed in the choroid plexus and cerebrospinal fluid of adult rats. Mol Endocrinol. (1989) 3:1559–68. 10.1210/mend-3-10-1559 2608049

[57] Yu H Mistry J Nicar MJ Khosravi MJ Diamandis A van Doorn J. Insulin-like growth factors (IGF-I, free IGF-I and IGF-II) and insulin-like growth factor binding proteins (IGFBP-2, IGFBP-3, IGFBP-6, and ALS) in blood circulation. J Clin Lab Anal. (1999) 13:166–72. 10.1002/(SICI)1098-2825(1999)13:4<166::AID-JCLA5>3.0.CO;2-X 10414596 PMC6808158

[58] Ho PJ Baxter RC Insulin-like growth factor-binding protein-2 in patients with prostate carcinoma and benign prostatic hyperplasia. Clin Endocrinol. (1997) 46:333–42. 10.1046/j.1365-2265.1997.1100922.x 9156044

[59] Juul A Dalgaard P Blum WF Bang P Hall K Michaelsen KF. Serum levels of insulin-like growth factor (IGF)-binding protein-3 (IGFBP-3) in healthy infants, children, and adolescents: the relation to IGF-I, IGF-II, IGFBP-1, IGFBP-2, age, sex, body mass index, and pubertal maturation. J Clin Endocrinol Metab. (1995) 80:2534–42. 10.1210/jcem.80.8.7543116 7543116

[60] Blum WF Horn N Kratzsch J Jørgensen JO Juul A Teale D. Clinical studies of IGFBP-2 by radioimmunoassay. Growth Regul. (1993) 3:100–4. 7683512

[61] Wolf E Kramer R Blum WF Foll J Brem G. Consequences of postnatally elevated insulin-like growth factor-II in transgenic mice: endocrine changes and effects on body and organ growth. Endocrinology. (1994) 135:1877–86. 10.1210/endo.135.5.7525257 7525257

[62] Reijnders CM Koster JG van Buul-Offers SC. Overexpression of human IGF-II mRNA in the brain of transgenic mice modulates IGFBP-2 gene expression in the medulla oblongata. J Endocrinol. (2004) 182:445–55. 10.1677/joe.0.1820445 15350186

[63] Chowen JA Goya L Ramos S Busiguina S Garcia SLM Argente J. Effects of early undernutrition on the brain insulin-like growth factor-I system. J Neuroendocrinol. (2004) 14:163–9. 10.1046/j.0007-1331.2001.00758.x 11849376

[64] Beilharz EJ Russo VC Butler G Baker NL Connor B Sirimanne ES. Co-ordinated and cellular specific induction of the components of the IGF/IGFBP axis in the rat brain following hypoxic-ischemic injury. Brain Res Mol Brain Res. (1998) 59:119–34. 10.1016/S0169-328X(98)00122-3 9729323

[65] Klempt M Klempt ND Gluckman PD. Hypoxia and hypoxia/ischemia affect the expression of insulin-like growth factor binding protein 2 in the developing rat brain. Brain Res Mol Brain Res. (1993) 17:347–50. 10.1016/0169-328X(93)90021-G 7685464

[66] Yao DL West NR Bondy CA Brenner M Hudson LD Zhou J. Cryogenic spinal cord injury induces astrocytic gene expression of insulin-like growth factor I and insulin-like growth factor binding protein 2 during myelin regeneration. J Neurosci Res. (1995) 40:647–59. 10.1002/jnr.490400510 7541476

[67] Walter HJ Berry M Hill DJ Logan A. Spatial and temporal changes in the insulin-like growth factor (IGF) axis indicate autocrine/paracrine actions of IGF-I within wounds of the rat brain. Endocrinology. (1997) 138:3024–34. 10.1210/endo.138.7.5284 9202248

[68] Sandberg NAC von HH Holmin S Sara VR Bellander BM Schalling M Increase of insulin-like growth factor (IGF)-1, IGF binding protein-2 and−4 mRNAs following cerebral contusion. Brain Res Mol Brain Res. (1996) 38:285–93. 10.1016/0169-328X(95)00346-T 8793117

[69] Gehrmann J Yao DL Bonetti B Bondy CA Brenner M Zhou J. Expression of insulin-like growth factor-I and related peptides during motoneuron regeneration. Exp Neurol. (1994) 128:202–10. 10.1006/exnr.1994.1128 8076663

[70] Jeong EY Kim S Jung S Kim G Son H Lee DH. Enhancement of IGF-2-induced neurite outgrowth by IGF-binding protein-2 and osteoglycin in SH-SY5Y human neuroblastoma cells. Neurosci Lett. (2013) 548:249–54. 10.1016/j.neulet.2013.05.038 23714241

[71] Shen F Song C Liu Y Zhang J Wei Song S. IGFBP2 promotes neural stem cell maintenance and proliferation differentially associated with glioblastoma subtypes. Brain Res. (2019) 1704:174–86. 10.1016/j.brainres.2018.10.018 30347220

[72] Basta KA Szczesny E Glombik K Slusarczyk J Trojan E Tomaszewski KA Prenatal stress leads to changes in IGF-1 binding proteins network in the hippocampus and frontal cortex of adult male rat. Neuroscience. (2014) 274:59–68. 10.1016/j.neuroscience.2014.05.010 24857711

[73] Li W Trovero F Cordier J Wang Y Drieu K Papadopoulos V. Prenatal exposure of rats to Ginkgo biloba extract (EGb 761) increases neuronal survival/growth and alters gene expression in the developing fetal hippocampus. Brain Res Dev Brain Res. (2003) 144:169–80. 10.1016/S0165-3806(03)00168-8 12935914

[74] Roet KC Franssen EH de Bree FM Essing AH Zijlstra SJ Fagoe ND. A multilevel screening strategy defines a molecular fingerprint of proregenerative olfactory ensheathing cells and identifies SCARB2, a protein that improves regenerative sprouting of injured sensory spinal axons. J. Neurosci. (2013) 33:11116–35. 10.1523/JNEUROSCI.1002-13.2013 23825416 PMC6618611

[75] Oka S Leon J Sakumi K Ide T Kang D LaFerla FM. Human mitochondrial transcriptional factor A breaks the mitochondria-mediated vicious cycle in Alzheimer's disease. Sci Rep. (2016) 6:37889. 10.1038/srep37889 27897204 PMC5126576

[76] Toledo JB Da X Bhatt P Wolk DA Arnold SE Shaw LM. Relationship between plasma analytes and SPARE-AD defined brain atrophy patterns in ADNI. PLoS ONE. (2013) 8:e55531. 10.1371/journal.pone.0055531 23408997 PMC3568142

[77] Hu WT Holtzman DM Fagan AM Shaw LM Perrin R Arnold SE. Plasma multianalyte profiling in mild cognitive impairment and Alzheimer disease. Neurology. (2012) 79:897–905. 10.1212/WNL.0b013e318266fa70 22855860 PMC3425844

[78] Lista S Faltraco F Prvulovic D Hampel H Blood and plasma-based proteomic biomarker research in Alzheimer's disease. Prog Neurobiol. (2013) 101–2:1–17. 10.1016/j.pneurobio.2012.06.007 22743552

[79] Lane EM Hohman TJ Jefferson AL. Insulin-like growth factor binding protein-2 interactions with Alzheimer's disease biomarkers. Brain Imaging Behav. (2017) 11:1779–86. 10.1007/s11682-016-9636-0 27817134 PMC5419882

[80] Chen RL Kassem NA Sadeghi M Preston JE. Insulin-like growth factor-II uptake into choroid plexus and brain of young and old sheep. J Gerontol A Biol Sci Med Sci. (2008) 63:141–8. 10.1093/gerona/63.2.141 18314448

[81] Castren E Ohga Y Berzaghi MP Tzimagiorgis G Thoenen H Lindholm D. bcl-2 messenger RNA is localized in neurons of the developing and adult rat brain. Neuroscience. (1994) 61:165–77. 10.1016/0306-4522(94)90069-8 7969891

[82] Klempt ND Klempt M Gunn AJ Singh K Gluckman PD. Expression of insulin-like growth factor-binding protein 2 (IGF-BP 2) following transient hypoxia-ischemia in the infant rat brain. Brain Res Mol Brain Res. (1992) 15:55–61. 10.1016/0169-328X(92)90151-Z 1279350

[83] Baker NL Carlo RV Bernard O D'Ercole AJ Werther GA. Interactions between bcl-2 and the IGF system control apoptosis in the developing mouse brain. Brain Res Dev Brain Res. (1999) 118:109–18. 10.1016/S0165-3806(99)00136-4 10611509

[84] Schwarz E Guest PC Rahmoune H Harris LW Wang L Leweke FM. Identification of a biological signature for schizophrenia in serum. Mol Psychiatry. (2012) 17:494–502. 10.1038/mp.2011.42 21483431

[85] Chan MK Krebs MO Cox D Guest PC Yolken RH Rahmoune H. Development of a blood-based molecular biomarker test for identification of schizophrenia before disease onset. Transl Psychiatry. (2015) 5:e601. 10.1038/tp.2015.91 26171982 PMC5068725

[86] Milanesi E Zanardini R Rosso G Maina G Barbon A Mora C. Insulin-like growth factor binding protein 2 in bipolar disorder: an expression study in peripheral tissues. World J Biol Psychiatry. (2018) 19:610–8. 10.1080/15622975.2017.1282172 28090803

[87] Benedetti F Poletti S Hoogenboezem TA Mazza E Ambree O de Wit H. Inflammatory cytokines influence measures of white matter integrity in Bipolar disorder. J Affect Disord. (2016) 202:1–9. 10.1016/j.jad.2016.05.047 27253210

[88] Lamers F Bot M Jansen R Chan MK Cooper JD Bahn S. Serum proteomic profiles of depressive subtypes. Transl Psychiatry. (2016) 6:e851. 10.1038/tp.2016.115 27404283 PMC5545705

[89] Kaufmann T van-der Meer D Doan NT Schwarz E Lund MJ Agartz I. Common brain disorders are associated with heritable patterns of apparent aging of the brain. Nat Neurosci. (2019) 22:1617–23. 10.1038/s41593-019-0471-7 31551603 PMC6823048

[90] Filiou MD Sandi C. Anxiety and brain mitochondria: a bidirectional crosstalk. Trends Neurosci. (2019) 42:573–88. 10.1016/j.tins.2019.07.002 31362874

[91] Schafer MJ White TA Iijima K Haak AJ Ligresti G Atkinson EJ. Cellular senescence mediates fibrotic pulmonary disease. Nat Commun. (2017) 8:14532. 10.1038/ncomms14532 28230051 PMC5331226

[92] Baker DJ Childs BG Durik M Wijers ME Sieben CJ Zhong J. Naturally occurring p16(Ink4a)-positive cells shorten healthy lifespan. Nature. (2016) 530:184–9. 10.1038/nature16932 26840489 PMC4845101

[93] Sim SE Chung YH Jeong JH Yun SW Lim HS Kim D. Immunohistochemical localization of insulin-like growth factor binding protein 2 in the central nervous system of SOD1(G93A) transgenic mice. J Mol Histol. (2009) 40:157–63. 10.1007/s10735-009-9219-0 19468844

[94] Liu Y Wang X Li W Zhang Q Li Y Zhang Z. A sensitized IGF1 treatment restores corticospinal axon-dependent functions. Neuron. (2017) 95:817–3.e4. 10.1016/j.neuron.2017.07.037 28817801 PMC5582621

[95] Ma KH Hung HA Srinivasan R Xie H Orkin SH Svaren J. Regulation of peripheral nerve myelin maintenance by gene repression through polycomb repressive complex 2. J Neurosci. (2015) 35:8640–52. 10.1523/JNEUROSCI.2257-14.2015 26041929 PMC4452560

[96] Arthur FPJ Latouche M Wilton DK Quintes S Chabrol E Banerjee A c-Jun reprograms Schwann cells of injured nerves to generate a repair cell essential for regeneration. Neuron. (2012) 75:633–47. 10.1016/j.neuron.2012.06.021 22920255 PMC3657176

[97] Ye P Carson J D'Ercole AJ. *In vivo* actions of insulin-like growth factor-I (IGF-I) on brain myelination: studies of IGF-I and IGF binding protein-1 (IGFBP-1) transgenic mice. J Neurosci. (1995) 15:7344–56. 10.1523/JNEUROSCI.15-11-07344.1995 7472488 PMC6578047

[98] Ye P Li L Richards RG DiAugustine RP D'Ercole AJ. Myelination is altered in insulin-like growth factor-I null mutant mice. J Neurosci. (2002) 22:6041–51. 10.1523/JNEUROSCI.22-14-06041.2002 12122065 PMC6757955

[99] Logan A Gonzalez AM Hill DJ Berry M Gregson NA Baird A. Coordinated pattern of expression and localization of insulin-like growth factor-II (IGF-II) and IGF-binding protein-2 in the adult rat brain. Endocrinology. (1994) 135:2255–64. 10.1210/endo.135.5.7525264 7525264

[100] Liu X Yao DL Bondy CA Brenner M Hudson LD Zhou J. Astrocytes express insulin-like growth factor-I (IGF-I) and its binding protein, IGFBP-2, during demyelination induced by experimental autoimmune encephalomyelitis. Mol Cell Neurosci. (1994) 5:418–30. 10.1006/mcne.1994.1052 7529631

[101] Chesik D De KJ Glazenburg L Wilczak N. Insulin-like growth factor binding proteins: regulation in chronic active plaques in multiple sclerosis and functional analysis of glial cells. Eur J Neurosci. (2006) 24:1645–52. 10.1111/j.1460-9568.2006.05034.x 17004928

[102] Ma KH Hung HA Svaren J. Epigenomic regulation of schwann cell reprogramming in peripheral nerve injury. J Neurosci. (2016) 36:9135–47. 10.1523/JNEUROSCI.1370-16.2016 27581455 PMC5005723

[103] Kuhl NM De KJ De VH Hoekstra D. Insulin-like growth factor binding proteins-1 and−2 differentially inhibit rat oligodendrocyte precursor cell survival and differentiation in vitro. J Neurosci Res. (2002) 69:207–16. 10.1002/jnr.10293 12111802

[104] Yao DL Liu X Hudson LD Webster HD. Insulin-like growth factor I treatment reduces demyelination and up-regulates gene expression of myelin-related proteins in experimental autoimmune encephalomyelitis. Proc Natl Acad Sci USA. (1995) 92:6190–4. 10.1073/pnas.92.13.6190 7541143 PMC41668

[105] Chesik D De KJ Wilczak N. Involvement of insulin-like growth factor binding protein-2 in activated microglia as assessed in post mortem human brain. Neurosci Lett. (2004) 362:14–6. 10.1016/j.neulet.2004.01.039 15147770

[106] Katz J Weiss H Goldman B Kanety H Stannard B LeRoith D. Cytokines and growth factors modulate cell growth and insulin-like growth factor binding protein secretion by the human salivary cell line (HSG). J Cell Physiol. (1995) 165:223–7. 10.1002/jcp.1041650202 7593199

[107] Streit WJ Walter SA Pennell NA. Reactive microgliosis. Prog Neurobiol. (1999) 57:563–81. 10.1016/S0301-0082(98)00069-0 10221782

[108] Bondy C Werner H Roberts CT Jr LeRoith D. Cellular pattern of type-I insulin-like growth factor receptor gene expression during maturation of the rat brain: comparison with insulin-like growth factors I and II. Neuroscience. (1992) 46:909–23. 10.1016/0306-4522(92)90193-6 1311816

[109] Chernausek SD Murray MA Cheung PT. Expression of insulin-like growth factor binding protein-4 (IGFBP-4) by rat neural cells–comparison to other IGFBPs. Regul Pept. (1993) 48:123–32. 10.1016/0167-0115(93)90341-5 7505459

[110] Burgess SK Jacobs S Cuatrecasas P Sahyoun N. Characterization of a neuronal subtype of insulin-like growth factor I receptor. J Biol Chem. (1987) 262:1618–22. 2948957

[111] Vajdos FF Ultsch M Schaffer ML Deshayes KD Liu J Skelton NJ. Crystal structure of human insulin-like growth factor-1: detergent binding inhibits binding protein interactions. Biochemistry. (2001) 40:11022–9. 10.1021/bi0109111 11551198

[112] Shemer J Raizada MK Masters BA Ota A LeRoith D. Insulin-like growth factor I receptors in neuronal and glial cells. Characterization and biological effects in primary culture. J Biol Chem. (1987) 262:7693–9. 2953724

[113] Heidenreich KA Freidenberg GR Figlewicz DP Gilmore PR. Evidence for a subtype of insulin-like growth factor I receptor in brain. Regul Pept. (1986) 15:301–10. 10.1016/0167-0115(86)90160-6 2948220

[114] Kiepe D Ulinski T Powell DR Durham SK Mehls O Tonshoff B. Differential effects of insulin-like growth factor binding proteins-1,−2,−3, and−6 on cultured growth plate chondrocytes. Kidney Int. (2002) 62:1591–600. 10.1046/j.1523-1755.2002.00603.x 12371959

[115] Gazit N Vertkin I Shapira I Helm M Slomowitz E Sheiba M. IGF-1 receptor differentially regulates spontaneous and evoked transmission via mitochondria at hippocampal synapses. Neuron. (2016) 89:583–97. 10.1016/j.neuron.2015.12.034 26804996 PMC4742535

[116] Azar WJ Zivkovic S Werther GA Russo VC. IGFBP-2 nuclear translocation is mediated by a functional NLS sequence and is essential for its pro-tumorigenic actions in cancer cells. Oncogene. (2014) 33:578–88. 10.1038/onc.2012.630 23435424

[117] Conover CA Johnstone EW Turner RT Evans GL John BFJ Doran PM. Subcutaneous administration of insulin-like growth factor (IGF)-II/IGF binding protein-2 complex stimulates bone formation and prevents loss of bone mineral density in a rat model of disuse osteoporosis. Growth Horm IGF Res. (2002) 12:178–83. 10.1016/S1096-6374(02)00044-8 12162999

[118] Fisher MC Meyer C Garber G Dealy CN. Role of IGFBP2, IGF-I and IGF-II in regulating long bone growth. Bone. (2005) 37:741–50. 10.1016/j.bone.2005.07.024 16183342

[119] DeMambro VE Maile L Wai C Kawai M Cascella T Rosen CJ. Insulin-like growth factor-binding protein-2 is required for osteoclast differentiation. J Bone Miner Res. (2012) 27:390–400. 10.1002/jbmr.545 22006816 PMC3385417

[120] Xi G Wai C DeMambro V Rosen CJ Clemmons DR. IGFBP-2 directly stimulates osteoblast differentiation. J Bone Miner Res. (2014) 29:2427–38. 10.1002/jbmr.2282 24839202 PMC5117190

[121] Xi G Solum MA Wai C Maile LA Rosen CJ Clemmons DR. The heparin-binding domains of IGFBP-2 mediate its inhibitory effect on preadipocyte differentiation and fat development in male mice. Endocrinology. (2013) 154:4146–57. 10.1210/en.2013-1236 23981772 PMC3800754

[122] Allen NJ Bennett ML Foo LC Wang GX Chakraborty C Smith SJ. Astrocyte glypicans 4 and 6 promote formation of excitatory synapses via GluA1 AMPA receptors. Nature. (2012) 486:410–4. 10.1038/nature11059 22722203 PMC3383085

[123] Fletcher L Isgor E Sprague S Williams LH Alajajian BB Jimenez DF. Spatial distribution of insulin-like growth factor binding protein-2 following hypoxic-ischemic injury. BMC Neurosci. (2013) 14:158. 10.1186/1471-2202-14-158 24359611 PMC3911968

[124] Warren MS Bradley WD Gourley SL Lin YC Simpson MA Reichardt LF. Integrin beta1 signals through Arg to regulate postnatal dendritic arborization, synapse density, and behavior. J Neurosci. (2012) 32:2824–34. 10.1523/JNEUROSCI.3942-11.2012 22357865 PMC3313657

[125] Newton SS Collier EF Hunsberger J Adams D Terwilliger R Selvanayagam E. Gene profile of electroconvulsive seizures: induction of neurotrophic and angiogenic factors. J Neurosci. (2003) 23:10841–51. 10.1523/JNEUROSCI.23-34-10841.2003 14645477 PMC6740983

[126] Burgdorf J Colechio EM Ghoreishi HN Gross AL Rex CS Zhang XL. IGFBP2 produces rapid-acting and long-lasting effects in rat models of posttraumatic stress disorder via a novel mechanism associated with structural plasticity. Int J Neuropsychopharmacol. (2017) 20:476–84. 10.1093/ijnp/pyx007 28158790 PMC5458343

[127] Suliman S Mkabile SG Fincham DS Ahmed R Stein DJ Seedat S. Cumulative effect of multiple trauma on symptoms of posttraumatic stress disorder, anxiety, and depression in adolescents. Compr Psychiatry. (2009) 50:121–7. 10.1016/j.comppsych.2008.06.006 19216888

[128] Malberg JE Platt B Rizzo SJ Ring RH Lucki I Schechter LE. Increasing the levels of insulin-like growth factor-I by an IGF binding protein inhibitor produces anxiolytic and antidepressant-like effects. Neuropsychopharmacology. (2007) 32:2360–8. 10.1038/sj.npp.1301358 17342171

[129] Stein TD Anders NJ DeCarli C Chan SL Mattson MP Johnson JA. Neutralization of transthyretin reverses the neuroprotective effects of secreted amyloid precursor protein (APP) in APPSW mice resulting in tau phosphorylation and loss of hippocampal neurons: support for the amyloid hyothesis. J Neurosci. (2004) 24:7707–17. 10.1523/JNEUROSCI.2211-04.2004 15342738 PMC6729623

[130] Rowe WB Blalock EM Chen KC Kadish I Wang D Barrett JE. Hippocampal expression analyses reveal selective association of immediate-early, neuroenergetic, and myelinogenic pathways with cognitive impairment in aged rats. J Neurosci. (2007) 27:3098–110. 10.1523/JNEUROSCI.4163-06.2007 17376971 PMC6672456

[131] Zhang JX Chen XQ Du JZ Chen QM Zhu CY. Neonatal exposure to intermittent hypoxia enhances mice performance in water maze and 8-arm radial maze tasks. J Neurobiol. (2005) 65:72–84. 10.1002/neu.20174 16010673

[132] Lu XJ Chen XQ Weng J Zhang HY Pak DT Luo JH. Hippocampal spine-associated Rap-specific GTPase-activating protein induces enhancement of learning and memory in postnatally hypoxia-exposed mice. Neuroscience. (2009) 162:404–14. 10.1016/j.neuroscience.2009.05.011 19442707 PMC3243647

[133] Refojo D Schweizer M Kuehne C Ehrenberg S Thoeringer C Vogl AM. Glutamatergic and dopaminergic neurons mediate anxiogenic and anxiolytic effects of CRHR1. Science. (2011) 333:1903–7. 10.1126/science.1202107 21885734

[134] White V Jawerbaum A Mazzucco MB Gauster M Desoye G Hiden U. Diabetes-associated changes in the fetal insulin/insulin-like growth factor system are organ specific in rats. Pediatr Res. (2015) 77:48–55. 10.1038/pr.2014.139 25268143

[135] Stranahan AM Arumugam TV Cutler RG Lee K Egan JM Mattson MP. Diabetes impairs hippocampal function through glucocorticoid-mediated effects on new and mature neurons. Nat Neurosci. (2008) 11:309–17. 10.1038/nn2055 18278039 PMC2927988

[136] Agrawal V Tee MK Qiao J Muench MO Miller WL. Potential role of increased oxygenation in altering perinatal adrenal steroidogenesis. Pediatr Res. (2015) 77:298–309. 10.1038/pr.2014.194 25470028

[137] Yakar S Domene H Meidan R Cassorla F Gilad I Koch I. Growth hormone (GH) stimulates insulin-like growth factor-I (IGF-I) and IGF-binding protein (IGFBP)-2 gene expression in spleens of juvenile rats. Horm Metab Res. (1994) 26:363–6. 10.1055/s-2007-1001707 7528707

[138] Cardona-Gomez GP Chowen JA Garcia-Segura LM. Estradiol and progesterone regulate the expression of insulin-like growth factor-I receptor and insulin-like growth factor binding protein-2 in the hypothalamus of adult female rats. J Neurobiol. (2000) 43:269–81. 10.1002/(sici)1097-4695(20000605)43:3<269::aid-neu5>3.0.co;2-d 10842239

